# ESPO/HEALTH SCIENCES: STRENGTH IN NUMBERS: LEVERAGING THE POWER OF NETWORKS FOR RECRUITING OLDER ADULTS AND CAREGIVERS

**DOI:** 10.1093/geroni/igz038.2196

**Published:** 2019-11-08

**Authors:** Karen O Moss, Anyah Prasad, Fayron Epps

**Affiliations:** 1 The Ohio State University, Columbus, United States; 2 University of Massachusetts Boston, Boston, Massachusetts, United States; 3 Georgia State University, Atlanta, Georgia, United States

## Abstract

Recruitment of older adults and/or their family caregivers for participation in research is challenging. This process is further complicated by population or procedure-specific factors that can impede recruitment efforts. The purpose of this symposium is to describe various recruitment challenges and solutions as well as strategies used to engage older adults and family caregivers in research. Dr. Glenna Brewster will present recruitment strategies used for a multisite telehealth intervention for caregivers of persons living with dementia. Dr. Todd Monroe will examine barriers and solutions for recruitment and retention of persons with Alzheimer’s disease for neuroimaging studies. Dr. Karen Moss will share strategies used to recruit family caregivers of African American older adults with dementia for end-of-life research. Dr. Shiva Prasad will share strategies to recruit LGBT older adults to a study on exploring the idea of an LGBT online senior center, and Dr. Karen Rose will describe stigma associated with recruiting persons with Alzheimer’s disease and other dementias, and their family caregivers, including best practices from the literature coupled with lessons learned from experiences. Shared strategies and solutions will assist researchers in identifying and addressing recruitment challenges and help to ensure recruitment of diverse groups of older adults and/or family caregivers for studies that use various research methodologies, including leveraging the power of professional and community-based networks. Increasing research participation will yield the knowledge necessary for improving health outcomes for older adults and their family caregivers living with serious chronic illnesses such as Alzheimer’s disease and related dementias.

